# Engineering Characteristics of Chemically Treated Water-Repellent Kaolin

**DOI:** 10.3390/ma9120978

**Published:** 2016-12-02

**Authors:** Youngmin Choi, Hyunwook Choo, Tae Sup Yun, Changho Lee, Woojin Lee

**Affiliations:** 1 School of Civil, Environmental, and Architectural Engineering, Korea University, Seoul 02841, Korea; junwi@korea.ac.kr (Y.C.); choohw@gmail.com (H.C.); 2 School of Civil and Environmental Engineering, Yonsei University, Seoul 03722, Korea; taesup@yonsei.ac.kr; 3 Department of Marine and Civil Engineering, Chonnam National University, Yeosu 59626, Korea; changho@jnu.ac.kr

**Keywords:** artificial water-repellent clay, contact angle, landfill cover system, organosilane, stiffness, water infiltration

## Abstract

Water-repellent soils have a potential as alternative construction materials that will improve conventional geotechnical structures. In this study, the potential of chemically treated water-repellent kaolin clay as a landfill cover material is explored by examining its characteristics including hydraulic and mechanical properties. In order to provide water repellency to the kaolin clay, the surface of clay particle is modified with organosilanes in concentrations (C_O_) ranging from 0.5% to 10% by weight. As the C_O_ increases, the specific gravity of treated clay tends to decrease, whereas the total organic carbon content of the treated clay tends to increase. The soil-water contact angle increases with an increase in C_O_ until C_O_ = 2.5%, and then maintains an almost constant value (≈134.0°). Resistance to water infiltration is improved by organosilane treatment under low hydrostatic pressure. However, water infiltration resistance under high hydrostatic pressure is reduced or exacerbated to the level of untreated clay. The maximum compacted dry weight density decreases with increasing C_O_. As the C_O_ increases, the small strain shear modulus increases, whereas the effect of organosilane treatment on the constrained modulus is minimal. The results indicate that water-repellent kaolin clay possesses excellent engineering characteristics for a landfill cover material.

## 1. Introduction

Landfill needs a final cover system to prevent the uncontrolled release of landfill gas and the infiltration of precipitated water into the waste [1,2]. Although conventional cover systems such as compacted clay liners, geomembranes, and geosynthetic clay liners have been successfully utilized [1,3], those systems may not provide a feasible solution in regions with arid climates [4,5]. Thus, the evapotranspiration cover system (ET) has been suggested as an alternative.

The ET cover system relies on the water storage capacity of the soil layer, rather than the hydraulic conductivity of the soil layer, to minimize the infiltration. The soil layer retains precipitated water until the water evaporates from the surface or transpires through vegetation [6]. Two general types of ET cover systems have been used: a monolithic barrier and a capillary barrier. A monolithic barrier uses a single layer of fine-grained soil such as silt or clayey silt, whereas a capillary barrier consists of a fine soil layer over a coarser layer. Although a capillary barrier can retain more water than a monolithic cover with equal thickness [6,7,8], the retained water can infiltrate relatively quickly into the body of the landfill when a fine-grained soil layer within a capillary barrier becomes fully saturated [9,10].

One possible technique to enhance the ET cover system is to employ water-repellent soil material as an infiltration barrier layer [11]. Since water on a hydrophobic surface forms distinct droplets, water-repellent soils can resist or retard water infiltration through the soil surface [12]. Water-repellent soil can be made by natural or anthropogenic processes, such as wildfire, microbial activity, exudates from living plants, decomposition of litter, oil spills, and cultivation of crops [13,14,15,16]. Although natural water-repellent soils are commonly found throughout the world [14], they may be unsuitable engineering materials because of highly anisotropic and heterogeneous water-repellent characteristics and the likelihood that natural water repellants will not persist for long. Therefore, artificially created water-repellent soils by using wax [17], organosilanes [18,19], and other hydrophobic agents [11,20] have recently been investigated because they exhibit homogeneous water repellency and it is easy to control the degree of water repellency. Because artificial water-repellent sandy soils can be used as alternative construction materials for several purposes, such as surfaces for horse racing tracks [17], waterproofing layer of highways [13], water harvesting [21], and landfill barrier systems [22,23,24], many studies have been performed to characterize the engineering properties of artificial water-repellent sands. In contrast, there is a lack of research on artificially treated water-repellent clays as a construction material.

Clayey soils have typically been used as barriers to control water infiltration because of their low permeability. Additionally, water-repellent soils are well suited for controlling infiltration. Consequently, in this study, the potential of water-repellent clay used as a landfill cover material is explored by assessing engineering characteristics including hydraulic and mechanical properties. Kaolin clay was chemically treated with different concentrations of organosilane solutions to produce samples with different degrees of water repellency. These samples were subjected to a series of hydraulic and mechanical experiments to measure the soil-water contact angle, water infiltration time, infiltration rate, compaction characteristics, compressibility, and small strain shear modulus.

## 2. Materials and Methods

### 2.1. Materials

**Figure 1:**
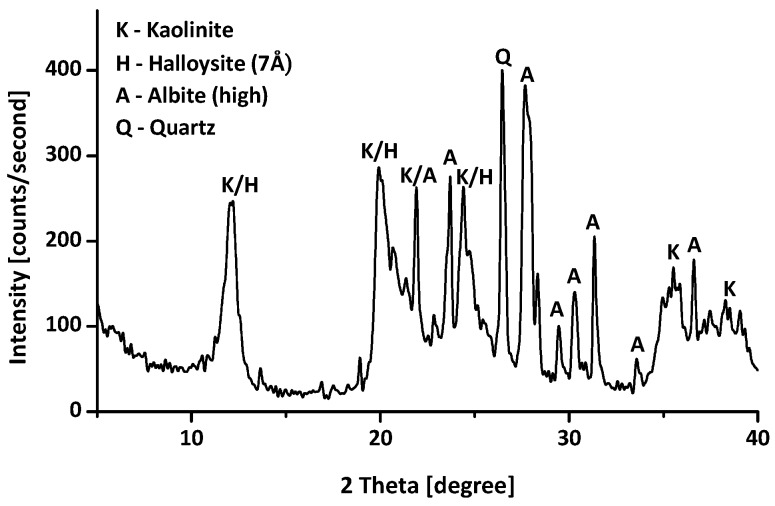
X-ray diffraction (XRD) pattern of the untreated kaolin clay.

The kaolin clay used in this study was purchased from Lakwoo Industry Co. Ltd., Gyeongnam Province, Korea. The mineralogy of the clay was assessed using X-ray diffraction (XRD) (Philips, X’Pert MPD, Almelo, The Netherlands). The clay consists mainly of kaolinite, with a minor amount of halloysite (Figure 1). The chemical composition of the clay was measured using X-ray fluorescence (XRF) spectrometry (Philips, PW2404, Almelo, The Netherlands). The majority of the chemical elements are silicon (Si) and aluminum (Al) as shown in Table 1. The specific gravity (G_s_), measured by a gas pycnometer (PMI, PYC-G100A-1, Ithaca, NY, USA), of the kaolin clay is 2.64, and the plastic and liquid limits of the soil are 23.6% and 35.9%, respectively. The median grain size (D_50_) of untreated kaolin is 10.35 μm. The specific surface area of the kaolin is 13.46 m^2^/g (methylene blue spot test) [25], and the pH value of the kaolin is 7.3 [26]. The total organic carbon (TOC) content, measured with a TOC analyzer (Shimadzu, TOC-VCPH, Kyoto, Japan), of the kaolin is 0.062% ± 0.012%. The untreated kaolin was classified as clay with low plasticity (CL), in accordance with the Unified Soil Classification System [27].

**Figure 2:**
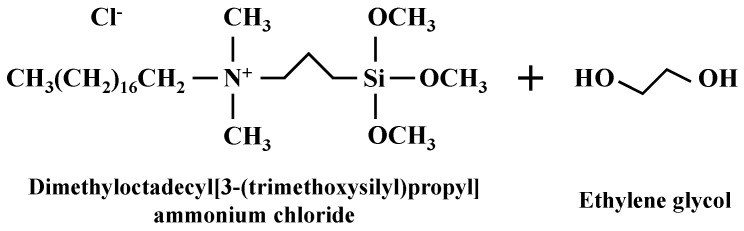
Chemical structure of the Zycosoil.

To create a water-repellent clay, kaolin clay was treated with an organosilane (Zycosoil, Zydex industries, Vadodara, India) in this study. Figure 2 shows the chemical structure of Zycosoil. The composition of Zycosoil is 40% 3-(trimethoxysilyl)propyl dimethyloctadecyl ammonium chloride (organosilane) and 60% ethylene glycol (solvent) [18,28]. When Zycosoil is diluted with water and mixed with soil, the hydrolyzable group forms siloxane bonds with the soil surface, and the non-hydrolyzable group imparts hydrophobicity to the soil surface [18,29]. Note that the XRD patterns of the treated clays are similar to the XRD pattern of untreated clay.

### 2.2. Preparation of Water-Repellent Clay

The water-repellent clay was prepared according to the method of Lee et al. [19]. The kaolin was thoroughly washed several times with deionized water to remove any contaminants and then dried in an oven (Hyundae Precision Industry, Seoul, Korea) for 24 h. 3 kg of the dried soil was mechanically blended with deionized water (6 L) for 24 h to ensure complete dispersion. In order to assign different degrees of water repellency, six different concentrations (C_O_) of Zycosoil (W_Zycosoil_/W_water_ = 0.5%, 0.75%, 1%, 2.5%, 5%, and 10%) were poured into the slurry (Table 2). After continuous stirring for 24 h, the treated clay was washed several times with deionized water to remove surplus organosilane and then dried in an oven for 24 h. Prior to experimentation, the oven-dried clay was ground with a mortar and pestle, and the ground soil was sieved with a #60 sieve.

### 2.3. Methods

Six different experiments were performed to explore the effect of water repellency on the following characteristics: soil-water contact angle, water infiltration time, water infiltration rate, compaction characteristics, constrained modulus, and small strain shear modulus.

#### 2.3.1. Soil-Water Contact Angle

Measurement of the solid-water contact angle is a direct method of assessing the degree of water repellency of a solid surface [30,31]. A homogeneous and absolutely flat surface is required to precisely measure the contact angle. Since it is impossible to obtain a sufficiently large flat area to measure a direct contact angle with a single soil particle, the sessile drop contact angle method (SDM) has been proposed [31].

One side of double-sided adhesive tape was attached to a slide glass, and then the oven-dried soil samples were sprinkled on the other side. The soil layer on the slide glass was compressed with a 100 g weight for 10 s, and then the slide was tapped carefully to remove unattached grains. After repeating this procedure twice, 10 drops of deionized water (2 ± 0.1 μL) were placed on the surface of the specimen with a micropipette (Axygen, AP-10, Corning, NY, USA) [31]. Horizontal images of the water droplets were captured within 5 s with a digital camera (Canon, PowerShot G9, Tokyo, Japan).

#### 2.3.2. Water Infiltration Time (WIT) and Infiltration Rate

The effect of water repellency and water pressure (WP) on infiltration time are explored by conducting an experiment as follows. Note that water infiltration time (WIT) is defined as the time until infiltrating water reaches the bottom of a specimen under a constant WP. To estimate the WIT, the soil samples were prepared in a cylindrical cell, 30 mm in inner diameter and 120 mm in height. The cell was made of polytetrafluoroethylene (PTFE) to prevent water from flowing between the soil sample and the wall [32]. Two electrodes and a filter paper at the bottom of the cell were used to determine the moment when infiltrated water wetted the paper. The soil was scooped into the cell and then tapped to achieve a constant porosity (n) of approximately 0.5 and a constant soil height of approximately 60 mm [33]. After the empty space on the soil in the cell was filled with deionized water, WP (10 kPa, 15 kPa, 20 kPa, or 30 kPa) was applied from the top using a pressure panel (Trautwein, M100000, Houston, TX, USA). Note the selected WPs in this study reflect the typical thickness of a fine-grained soil layer of ET cover systems (i.e., typically ranging from 0.4 m to 3 m) [7].

After the infiltrating water reaches the bottom of a specimen, the volume of outflow was measured over time to calculate the infiltration rate. Note that the infiltration rate was calculated for a given soil sample once a steady outflow was achieved [19]. The specimen was then extracted from the cell to measure the water content for estimating the degree of saturation (S).

#### 2.3.3. Compaction Characteristics

The compaction characteristics of the treated specimens were evaluated using a standard compaction mold (101.6 mm in diameter, Hyundae Precision Industry, Seoul, Korea) and a standard rammer (24.5 N, Hyundae Precision Industry, Seoul, Korea) [34]. Since the water-repellent clays were not easily wetted, the soil samples were prepared by thoroughly mixing with a deionized water for 30 min and then stored in a sealed plastic bag for 24 h before the compaction test. This procedure assures a more homogenous distribution of water in the water-repellent soil specimens [35,36]. The wetted soil was placed in three layers into the mold and each layer was compacted by 25 blows of the rammer with a falling height of 305 mm. The weight of the compacted soil was measured, and then three soil samples were obtained from the top, middle, and bottom of the specimens in order to determine the water content. This compaction procedure was repeated for each soil sample to establish compaction curves. Compaction characteristics, such as optimum volumetric water content (θ_opt_) and maximum dry unit weight (γ_d(max)_), were determined from the compaction curves.

#### 2.3.4. Constrained Modulus and Small Strain Modulus under K_0_ Loading Conditions

The constrained modulus and small strain modulus of the tested materials were explored using a zero-lateral strain (K_0_) oedometer cell [37]. The oedometer cell, made of brass, is 74 mm in diameter and 63 mm in height, with a wall thickness of 16 mm. The bender elements were installed in the top cap and bottom plate to measure a shear wave velocity (V_s_) of specimens. The soil samples were made of five layers. The dry soil was carefully scooped into the cell and densified by a static load of 7 kg within 10 s. The initial height of the soil specimens was approximately 45 mm. The vertical effective stress was incrementally doubled during each of the six loading steps until the vertical effective stress reached 625 kPa. Each loading step lasted for 1 h. Note that the rate of settlement prior to applying the next loading step was less than 0.001 mm/min. Settlement was measured using a linear variable differential transformer (LVDT) transducer (Macro sensors, GHSE 750-500, repeatability error <0.6 μm, Pennsauken Township, NJ, USA) with a DC power supply (Agilent, E3634A, Santa Clara, CA, USA) and a data logger (Agilent, 34970A, Santa Clara, CA, USA). In addition, the shear waves were measured at the end of each loading step using the following electronic peripherals. A signal generator (Agilent, 33220A, Santa Clara, CA, USA) provided a single sinusoidal signal as an input to the source bender element. The shear wave emitted from the source and propagated through the specimen was detected at the receiver bender element. The signal from the receiver was filtered and amplified by a filter amplifier (Krohn-Hite, 3364, Brockton, MA, USA). The filtered and amplified signal was digitized by an oscilloscope (Agilent, 54624A, Santa Clara, CA, USA). A total of 1024 signals were averaged to eliminate random noise. The shear wave velocity was calculated from the traveling distance, which is the tip-to-tip distance between the bender elements, and the first arrival time. The first arrival was identified from the recorded signal taking into consideration the near-field effect [38].

## 3. Experimental Results and Discussion

### 3.1. SEM and EDX Analysis of Tested Water-Repellent Kaolin

**Figure 3:**
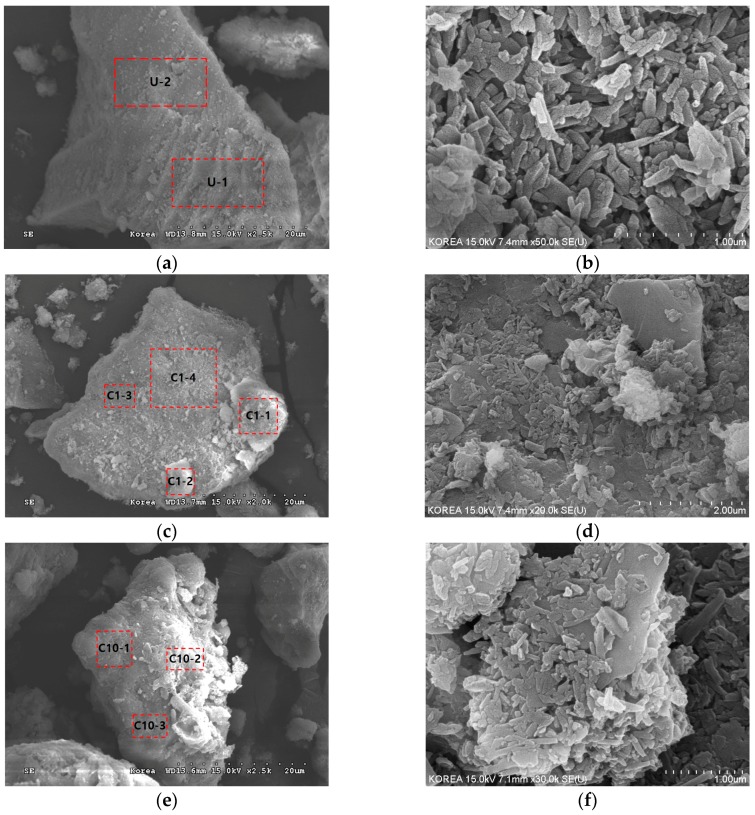
Field emission scanning electron microscopic (FESEM) images of untreated and treated soils with different concentrations (C_O_) of organosilane solution: (**a**,**b**) untreated; (**c**,**d**) C_O_ = 1%; and (**e**,**f**) C_O_ = 10%. Note that the energy dispersive X-ray (EDX) analysis was conducted on the marked areas in the figure, and the EDX results are tabulated in Table 3.

To examine the grafted pattern of organosilanes after treatment, field emission scanning electron microscopic (FESEM) and energy dispersive X-ray (EDX) analyses were carried out using a Hitachi S-4300 instrument. Figure 3 shows selected FESEM images of untreated and treated clays at C_O_ = 1% and 10%, and Table 3 shows EDX results of the marked areas in Figure 3. As shown in Figure 3a, angular and flat shaped kaolin particles are observed. The EDX analysis of untreated clay (e.g., area U-1 and U-2 in Table 3) reveals that the majority of its chemical elements are oxygen (O), aluminum (Al), and silicon (Si), with some minor elements including sodium (Na) and calcium (Ca). In the case of the treated clays, organosilanes grafted onto the particle surfaces can be seen in the FESEM images (e.g., area C1-1 and C1-2 in Figure 3c, and area C10-2 in Figure 3e). Note that the silane-grafted areas are brighter than the non-grafted areas in the FESEM images because of the difference in the mass intensity of the coated platinum. Irregular spatial distribution patterns of grafted silanes are evident in Figure 3c–f. Some areas are covered with a large amount of silanes with amalgamated structures, as shown in areas C1-1 and C1-2, while others are covered with fewer silanes, as in area C1-4, or are not coated such as areas of C1-3, C10-1, and C10-3. The irregular spatial distribution patterns of grafted silanes would be related to heterogeneous surface minerals (Figure 3b) due to presence of low and high surface energy sites of the mineral, because the silylation relies on the reactivity of the clay mineral surface [39]. As the C_O_ increases, the treated clay is covered with a greater amount of silanes with amalgamated structures, such as in area C10-2. The EDX results for treated clay (Table 3) show the presence of additional chemical elements, as compared with the untreated clay. Since the organosilane contains organic carbon molecules (Figure 2), carbon (C) is the main addition to the treated clay, while minor chemical elements (such as magnesium (Mg) and iron (Fe)) are also added. As the C_O_ increases from 0% to 1%, the atomic percentage of carbon in the particle surface increased to 11.90%–16.88%. Additionally, the atomic percentage of carbon in the treated clay with C_O_ = 10% is 21.84%. Note that the different silane grafting pattern shown in Figure 3c,e also clearly supports the difference in the concentration of carbon within grafted silanes.

### 3.2. Effect of Organosilane Concentration on G_s_, TOC, and Soil-Water Contact Angle

Grafting organosilane onto a soil particle may change the index properties of the soil, such as specific gravity (G_s_), particle size, TOC, and Atterberg limits, although relevant findings have rarely been reported until now [11,40]. To explore the effects of grafting organosilane on the index properties of soil, the G_s_ and TOC of water-repellent clay were measured.

**Figure 4:**
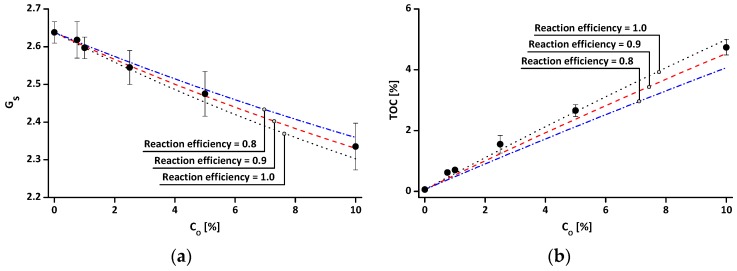
Effect of organosilane treatment concentration (C_O_) on the index properties: (**a**) specific gravity (G_s_); and (**b**) total organic carbon (TOC). Note that the error bars in the figures indicate standard deviations of measured values.

Figure 4a shows the variation in G_s_ of both untreated and treated soils according to C_O_. Since the G_s_ of the organosilane (=0.89 in this study) are much lower than that of the untreated kaolin (=2.638), the G_s_ of the treated soil decreases as C_O_ increases (Figure 4a). The G_s_ value of organosilane grafted soil (G_s_prediction_) can be predicted as follows:

(1) $$G_{s\_\mathrm{prediction}}=\frac{M_{\mathrm{soil}}+M_{\mathrm{added}\;\mathrm{silanes}}\times \mathrm{reaction}\;\mathrm{efficiency}}{\gamma_{w}\left(V_{\mathrm{soil}}+V_{\mathrm{added}\;\mathrm{silanes}}\times \mathrm{reaction}\;\mathrm{efficiency}\right)}$$

where γ_w_ is the density of water (g/cm^3^), M_soil_ (or V_soil_), and M_added silanes_ (or V_added silanes_) are the mass (or volume) of untreated soil and added silanes in the reaction solution, respectively. G_s_ values show a relatively good agreement with the values of G_s_prediction_ at a reaction efficiency of 0.9. Figure 4b shows the measured TOC values as a function of C_O_. Note that inorganic carbons are not detected in either the untreated or treated clays. Since the organosilane contains organic carbons, the TOC of the treated soil increases with increasing C_O_. The TOC value of treated soil (TOC_prediction_) can be predicted, as in Equation (2) below, with the assumptions that all of the carbon elements in the organosilane are regarded as organic carbon and there are no reactions between organosilane molecules.

(2) $${\mathrm{TOC}}_{\mathrm{prediction}}=\frac{M_{\mathrm{TOC}\;\mathrm{of}\;\mathrm{soil}}+M_{\mathrm{TOC}\;\mathrm{of}\;\mathrm{added}\;\mathrm{silanes}}\times \mathrm{reaction}\;\mathrm{efficiency}}{M_{\mathrm{soil}}+M_{\mathrm{added}\;\mathrm{silanes}}\times \mathrm{reaction}\;\mathrm{efficiency}}$$

where M_TOC of soil_, M_TOC of grafted silanes_, and M_TOC of added silanes_ are the mass of total organic carbon in the untreated soil, grafted silanes on the soil particles, and added silanes in the reaction solution, respectively. The measured TOC values were similar to the TOC_prediction_ with an input of reaction efficiency = 0.9–1.0. These analyses (Figure 4) reveal that the efficiency of silylation, in this study, is about 0.9–1.0. Also, the almost constant reaction efficiency with increasing C_O_ in Figure 4 indicates that: (1) the mass of grafted organosilanes on clay particles almost linearly increases with increasing C_O_; and (2) the end point of silylation is not reached until C_O_ = 10%.

**Figure 5:**
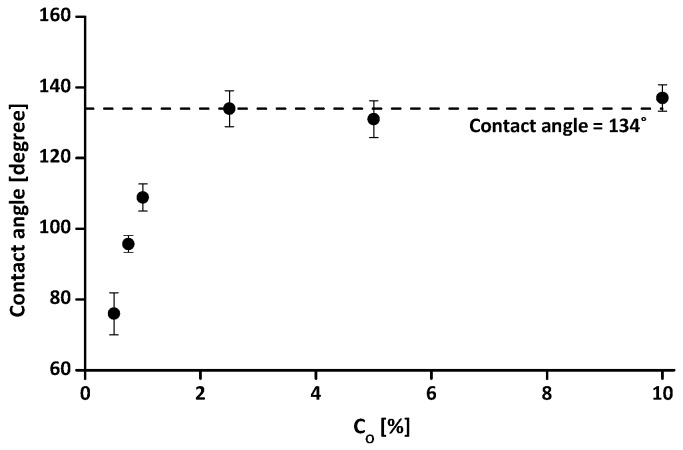
The measured soil-water contact angles with different treatment concentrations (C_O_). Note that the error bars in the figure indicate standard deviations of measured values from 10 water droplets.

Figure 5 presents the soil-water contact angles of the tested water-repellent clays with an increase in C_O_ from 0.5% to 10%. The soil-water contact angles tend to increase with increasing C_O_ until C_O_ = 2.5%, and then remain almost constant (≈134.0°) with a further increase in C_O_. Previous studies [20,41] also observed a similar trend, and Johnson and Dettre [42] reported that the advancing contact angle is almost constant when the coverage of non-wettable regions exceeds 40% of the total surface based on theoretical analysis for an idealized heterogeneous surface. Note a contact angle measured by SDM is often regarded as an advancing contact angle [43]. Consequently, the almost constant values of the measured contact angle in Figure 5 may imply that the clay particles were sufficiently covered with organosilanes when the C_O_ is approximately 2.5%. Note that a constant contact angle does not imply a constant amount of grafted organosilanes and degree of water repellency with further increase in C_O_.

### 3.3. Water Infiltration Time (WIT) and Water Infiltration Rate

The infiltration is mainly governed by two forces such as capillary and gravity forces [44]. Note that the value of capillary force would be changed from negative value (i.e., hydrophilic soil) to positive value (i.e., hydrophobic soil) when the soil was treated by organosilane. Therefore, the phenomenon of water infiltration is significantly related to hydrostatic water pressure (WP) on the soil surface and soil properties such as porosity, fabric, and degree of water repellency [33,45]. If the WP is higher than the water-entry pressure (WEP) of the soil, then water immediately penetrates into the soil. In contrast, water permeation is delayed when the WP is lower than the WEP of the soil. The time for water infiltration into the soil is an important factor in estimating the hydrological consequences of rain [46], and the time is mainly affected by the degree of water repellency of the soil [47] and WP [33]. However, the effects of water repellency and WP on the time required for water infiltration are not yet fully understood.

**Figure 6:**
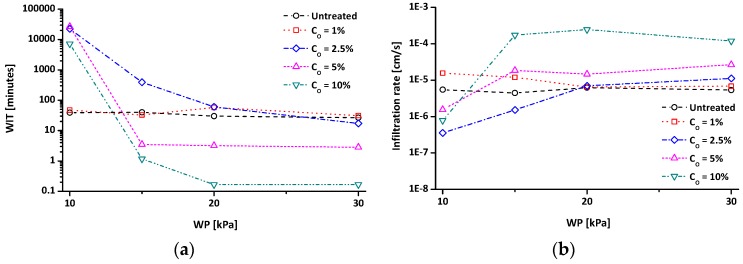
Effects of organosilane treatment concentration (C_O_) and applied hydrostatic water pressure (WP) on (**a**) the water-infiltration time (WIT); and (**b**) the infiltration rate during WIT experiment.

Figure 6 presents the variation of WIT and infiltration rate of tested materials with various C_O_ and the WP values under similar porosities (*n* ≈ 0.5). As the WP increases from 10 kPa to 30 kPa, the WIT of the untreated clay slightly decreases from ~39 min to ~27 min. The WIT of the C_O_ = 1% sample is similar to that of untreated clay. Note that the C_O_ = 1% sample shows a very high WIT value (>2 months) at WP of 5 kPa, although the data is not included in Figure 6a. In contrast, the WITs of the other treated clays are dramatically altered with changes in WP. The WITs of the treated clays with C_O_ = 2.5%, 5%, and 10% under WP = 10 kPa are 22,437 min, 26,888 min, and 7174 min, respectively. However, as the WP increases, the WIT of the C_O_ = 2.5% sample decreases and converges with the WIT of untreated clay, whereas the WITs of the C_O_ = 5% and 10% samples significantly decrease below the WIT of untreated clay.

The variation of the infiltration rate according to the applied WP can be seen in Figure 6b. The untreated clay shows an almost constant infiltration rate (4.44 × 10^−6^–6.28 × 10^−6^ cm/s), regardless of WP. The infiltration rate of the C_O_ = 1% specimen at 10 kPa is slightly higher than that of the untreated clay, and then it gradually reaches the level of the untreated soil. The C_O_ = 2.5%, 5%, and 10% specimens under 10 kPa pressure have a lower infiltration rate than that of untreated clay. As applied WP increases, the infiltration rates of these specimens tend to increase. The infiltration rate of the C_O_ = 2.5% specimen gradually approaches the infiltration rate of untreated clay, whereas the infiltration rates of the C_O_ = 5% and 10% specimens are higher than that of the untreated specimen at high WP.

The results of WIT and infiltration rate in Figure 6 are significantly related to the wetting patterns. Note that the wetting patterns of soil can be divided into two types: stable and unstable flows. When a soil is wettable, as is the case with untreated clay, a stable flow is observed with a uniform wetting front. Therefore, the wettable soil shows a high degree of saturation (S). In contrast, when a soil is non-wettable, as is the case with treated clays, an unstable flow is observed with an irregular and finger-like wetting front [48,49]. Therefore, the S value of non-wettable soil is low. Because the wetting front of tested specimens cannot be observed during WIT experiments, the S values of a disassembled specimen are employed as an indicator of the wetting patterns of the tested materials in this study.

**Figure 7:**
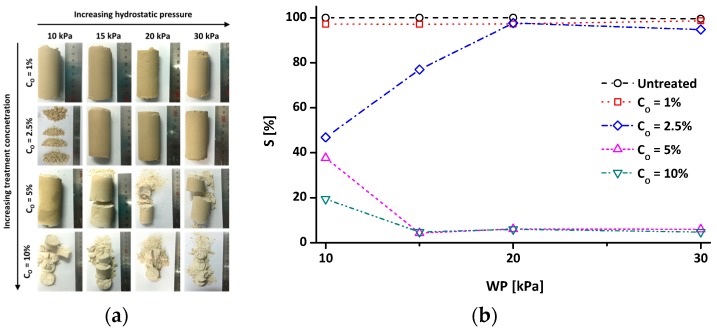
After a water infiltration time (WIT) experiment, (**a**) disassembled specimens; and (**b**) resulting degree of saturation (S) of the specimens. Note that the C_O_ = 2.5% sample at 10 kPa was extracted at depth by excavation because it is hard to maintain its original shape during a sample extraction from the cell.

Figure 7a,b shows representative pictures of disassembled specimens and their S values, respectively. Note that the C_O_ = 2.5% specimen at 10 kPa was extracted by excavating because it was difficult to maintain its original shape during sample extraction from the cell. The experimental results indicate that a specimen with uniform wetting exhibits a high S (>95%), whereas a specimen with irregular wetting exhibits a low S (<77%). The treated clays can be divided into two types according to the variation of S with WP: (1) increasing S with an increasing applied WP (Type-I, such as in the C_O_ = 2.5% specimen); and (2) decreasing S with an increasing applied WP (Type-II, such as in the C_O_ = 5% and 10% specimens). It is reasonable to infer that the effect of water repellency on infiltration vanishes for Type-I soil when the WP exceeds WEP as reflected in very high S values in Figure 7. Thus, the WIT and infiltration rate of the specimen with C_O_ = 2.5% approach to those of untreated kaolin (Figure 6). However, for Type-II soil, it seems that the water-repellent effect is not vanished even though the WP exceeds WEP. The low S values and the disassembled specimens in dry conditions (Figure 7) reflect the formation of finger-like unstable flow for Type-II soil when the WP exceeds WEP. In other words, the preferential flow pathways are formed for Type-II soil, so that the tested specimens with C_O_ = 5% and 10% show very low S values due to the limited saturation along the flow pathways. Because water can flow easily through the preferential flow pathways, the infiltration rate of water-repellent soils can be greater than that of wettable (or untreated) soil, in case finger-like flows are formed [48]. Thus, the specimens with C_O_ = 5% and 10% show a lower WIT or higher infiltration rate than those of untreated kaolin (Figure 6).

### 3.4. Compaction Characteristics

**Figure 8:**
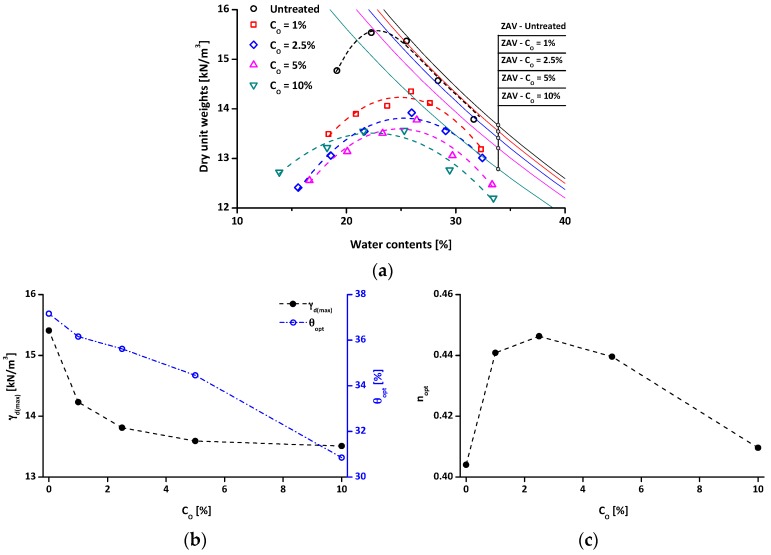
Compaction results: (**a**) compaction curves with zero air voids (ZAV) curves; (**b**) maximum dry unit weights (γ_d(max)_) and optimum volumetric water contents (θ_opt_); and (**c**) porosity at γ_d(max)_ (n_opt_) with the treatment concentration (C_O_).

The compaction curves (dotted lines) of the untreated and treated clays with zero air void (ZAV) curves (solid lines) are plotted in Figure 8a. The compaction curves of the treated soils are located below the compaction curve of untreated soil. This may be related to the combined effects of decreases both in G_s_ and in the degree of saturation of the water-repellent soils. The G_s_ reduction by organosilane treatment (Figure 4a) would induce a decrease in dry unit weight at a given porosity. The compaction curve of untreated clay approached the ZAV curve as the water content increased because the clay can be fully saturated [50]. On the other hand, the treated clays show a significant gap between their compaction curves and ZAV curves even though the specimens have a high water content, reflecting that it is hard to achieve full saturation of treated clays during compaction.

The γ_d(max)_ and θ_opt_ are calculated from the compaction curve, and plotted as a function of C_O_ in Figure 8b. Note that the mass-based water content is typically considered the optimal water content because of the ease of application in the construction field. However, since the mass-based water content is affected by the G_s_ of soil, the θ_opt_ is adopted to eliminate the effect of the different G_s_ values of treated clays on mass-based water content. As the C_O_ increases, the γ_d(max)_ exponentially decreases, whereas the G_s_ of the treated sample almost linearly decreases (Figure 4), and the θ_opt_ decreases in an approximately linear manner.

To estimate the effect of the organosilane treatment on the compaction, the porosity at γ_d(max)_ (n_opt_), as shown in Figure 8c, is computed by using the G_s_ and the γ_d(max)_ (i.e., n_opt_ = 1 − (γ_d(max)_/(G_s_∙γ_w_))). Note that, if the organosilane treatment does not affect compaction of soil, the n_opt_ of the treated samples will be theoretically the same as that of the untreated. The n_opt_ increases with increasing C_O_ until C_O_ = 2.5%, and then it decreases with a further increase in C_O_. Additionally, all of the n_opt_ values for the treated samples are higher than that of the untreated sample. This observation reflects that the organosilane treatment disrupts to achieve a dense state of specimen during the compaction test.

The relation between the n_opt_ and the C_O_ is presumably related to the combined effect of water repellency and friction resistance of particle contact. During the compaction test, water acts as a lubricant by developing water films around particles [51]. However, for the treated samples, the water repellency will disrupt the development of water films around particles and induce a consequently high n_opt_. Since the measured contact angle tend to increase with increasing C_O_ until C_O_ = 2.5%, and then remain almost constant (Figure 5), the resulting n_opt_ will increase as C_O_ increases to 2.5%. In contrast, the friction resistance of particle contact will be decreased with an increase in C_O_ [19,52], and the reduction in the friction resistance will promote achievement of a lower n_opt_ with increasing C_O_. Therefore, for C_O_ ≥ 2.5%, the n_opt_ decreases with increasing C_O_.

In summary, the compaction characteristics of treated clays are considerably different from those of untreated clay in terms of water repellency, G_s_ of the soil, and friction of particle contact. Those factors consequently induce a low value of γ_d(max)_. Based on the compaction test results, water-repellent clay has some advantages as a cap material for landfill because of its light weight.

### 3.5. Compressibility and Small Strain Shear Modulus

**Figure 9:**
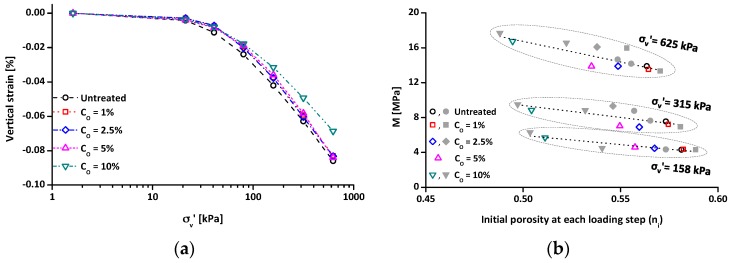
Zero-lateral strain oedometer experiment results: (**a**) stress-strain curves; and (**b**) constrained modulus (M) with initial porosity at each loading step (n_i_). Note that data (closed symbols in Figure 9b) indicate the M values of the untreated and treated clay specimens with various initial porosities due to different tamping energies.

To evaluate the compressibility characteristics of the treated clays, a one-dimensional oedometer experiment was conducted. As shown in Figure 9a, all stress-strain curves of the treated clays are located slightly above the stress-strain curve of untreated clay. The treated clays (C_O_ = 1%, 2.5%, and 5%) have almost the same stress-strain curves, whereas the C_O_ = 10% specimen shows lower settlement than the others. Note that the initial porosities of the dry tested specimens, which were compacted with the same compaction energy, decrease with increasing C_O_, because organosilane grafting onto the soil surface encourages a dense packing of soil [19,52,53]. Hence, the compressibility characteristic (constrained modulus (M)) of the tested specimen is plotted with the initial porosity at each loading step (n_i_) (Figure 9b). Note that M (= *d*σ’_v_/*d*ε_v_) is the slope of a compression curve where the *x*-axis is vertical strain (ε_v_) and the *y*-axis is vertical effective stress (σ’_v_). Figure 9b shows the M values of the tested specimens with varying initial porosities under three different σ’_v_ values. It can be observed in Figure 9b that, with an increase in applied σ’_v_, the M is increased due to the non-linear stress-strain behavior of soils (Figure 9a). Additionally, Figure 9b demonstrates that, under the same σ’_v_, the M value tends to increase with a decrease in porosity. Most notably, consistent with the previous studies [54,55], the M values of the tested materials can be a single function of the initial porosity (Figure 9b). These results imply that the effect of organosilane treatment on the compressibility is insignificant.

**Figure 10:**
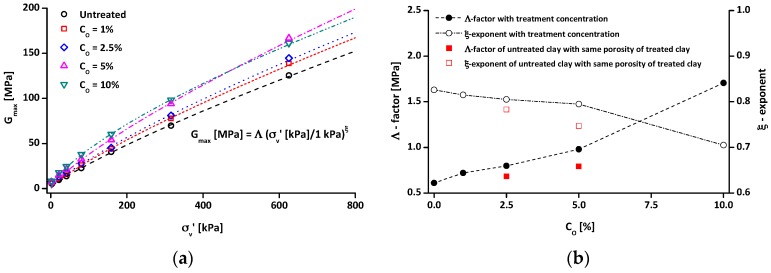
Small strain property of water-repellent clay: (**a**) shear modulus at small strain (G_max_); and (**b**) experimental determined factor (Λ-factor) and exponent (ξ-exponent) of the shear modulus. Note that the rectangular symbols (■ and □) indicate the Λ-factor and ξ-exponent of untreated clay under the same initial porosities of the C_O_ = 2.5% and C_O_ = 5% specimens.

Figure 10 presents the relation between G_max_ and vertical effective stress. Since the densities of each specimen were different, the shear modulus at small strain (G_max_) is used, instead of the shear wave velocity (V_s_), to explore the effects of organosilane treatment on small strain properties. Note that the G_max_ depends on the stiffness of inter-particle contacts and the inter-particle coordination, and it can be expressed as follows:

(3) $$G_{\max}={\rho V}_{s}^{2}=\Lambda {\left(\frac{\sigma_{v}^{\prime }}{\mathrm{kPa}}\right)}^{\xi }$$

where ρ is mass density, Λ and ξ are experimentally determined factors. Λ is the value of the G_max_ at 1 kPa, and ξ is the stress sensitivity of G_max_ with $\sigma_{v}^{\prime }$. Generally, stiffer materials show greater Λ, but smaller ξ [56]. As shown in Figure 10a, G_max_ increases with increasing C_O_, although the G_max_ of the C_O_ = 5% sample at 625 kPa is slightly higher than the G_max_ of the C_O_ = 10% sample. Λ and ξ are plotted with C_O_ in Figure 10b. It can be observed that the Λ factor increases with C_O_, whereas the ξ exponent decreases with an increase in C_O_, reflecting an increase in the stiffness of tested soils. Note that the initial porosity of the tested specimens decreases with an increase in C_O_; therefore, the tested materials with high C_O_ will have higher inter-particle coordination (or better contacts between particles). This results in an increased G_max_ of tested materials with an increase in C_O_.

To isolate/minimize the effect of the changed inter-particle coordination (or porosity) due to the grafting of organosilane on G_max_ of tested soils, an untreated clay specimen was subjected to high compaction energy to achieve the same initial porosities of the treated clays (C_O_ = 2.5% and 5% specimens). The filled and open rectangular symbols in Figure 10b indicate the Λ factor and the ξ exponent of the untreated clay, respectively. Although the initial porosity of the untreated clay is the same as that of the treated clay, the untreated clay shows lower Λ factor and ξ exponent values than the treated clay, and the difference between the untreated clay and the treated clay increases with C_O_. This reflects that the organosilane treatment enhances the stiffness (G_max_) of tested kaolin at a given porosity. Because the comparison of G_max_ under the same porosity may guarantee very similar inter-particle coordination, this increased G_max_ with an increase in C_O_ may be attributed to the increased inter-particle contact stiffness. In other words, at small strain level, the contacts between kaolin and organosilane, or between organosilane and organosilane, could be stiffer than those between kaolin and kaolin.

## 4. Conclusions

In this study, the hydraulic and mechanical characteristics of chemically-treated water-repellent clay are investigated to explore its usefulness as an alternative landfill cover material. A series of laboratory experiments was conducted to measure G_s_, TOC, soil-water contact angle, WIT, infiltration rate, compaction characteristics, compressibility, and small-strain shear modulus. The results of this study demonstrate the following:

1. The G_s_ and the TOC of treated clay indicate that the mass of grafted organosilanes on clay particles almost linearly increases with increasing C_O_. The maximal soil-water contact angle of the treated clay is achieved when the C_O_ is approximately 2.5%.
2. The treated clays can be divided into two types according to the variation of S with WP: (1) Type-I soil shows an increase in S values with increasing applied WP; and (2) Type-II soil shows a decrease in S with increasing applied WP. Both Type-I and Type-II soils show a superior performance as an infiltration barrier compared with untreated kaolin. However, Type-II soil (treated with C_O_ ≥ 5%) exhibits lower WIT values and a higher infiltration rate than untreated clay at high WP due to the formation of finger-like unstable flow.
3. The compaction characteristics of tested materials are affected by the organosilane treatment due to the combined effects of water repellency, G_s_ of the soil, and friction of particle contact, resulting in the treated clay showing a decreased γ_d(max)_. The effect of organosilane treatment on the M is minimal, whereas the G_max_ is increased with an increase in C_O_.
4. The findings of this study reveal that water-repellent clay (i.e., Type-I soil) has a potential to be a landfill cover material. Future work should consider the use of other hydrophobic agents to enhance in WEP of treated soil and the infiltration resistance of layered systems, such as a capillary barrier, to investigate optimum composition and soil layer thicknesses of the cover system.

## Figures and Tables

**Table 1:** X-ray fluorescence analysis of the untreated kaolin.

Component	SiO_2_	Al_2_O_3_	CaO	Na_2_O	Fe_2_O_3_^1^	K_2_O	TiO_2_	MnO	P_2_O_5_	LOI ^2^
wt.% composition	47.66	34.01	5.51	1.72	1.32	0.48	0.16	0.01	0.01	8.74

^1^ Fe_2_O_3_ = total Fe; ^2^ Loss of ignition.

**Table 2:** Preparation of water-repellent clay.

Sample	Kaolin (g)	Water (mL)	Zycosoil (g)
C_O_ = 0.5%	3000	6000	30
C_O_ = 0.75%			45
C_O_ = 1%			60
C_O_ = 2.5%			150
C_O_ = 5%			300
C_O_ = 10%			600

**Table 3:** Energy dispersive X-ray analysis results.

Atomic (%)	U-1	U-2	C1-1	C1-2	C1-3	C1-4	C10-1	C10-2	C10-3
C	-	-	11.90	16.88	-	12.48	-	21.84	-
O	66.63	66.93	63.90	60.86	67.39	63.02	64.08	57.94	62.98
Al	10.38	10.87	11.60	10.08	12.09	10.07	16.41	9.47	16.81
Si	17.06	16.06	12.60	12.18	16.78	12.59	19.51	10.75	20.21
Na	2.65	2.70	-	-	-	-	-	-	-
Mg	-	-	-	-	1.06	-	-	-	-
Ca	3.28	3.43	-	-	-	-	-	-	-
Fe	-	-	-	-	2.68	1.84	-	-	-
